# Pharmacokinetics, Pharmacodynamics, and Side Effects of Midazolam: A Review and Case Example

**DOI:** 10.3390/ph17040473

**Published:** 2024-04-08

**Authors:** Jens-Uwe Peter, Peter Dieudonné, Oliver Zolk

**Affiliations:** 1 Institute of Clinical Pharmacology, Immanuel Klinik Rüdersdorf, Brandenburg Medical School, 15562 Rüdersdorf, Germany; jens-uwe.peter@mhb-fontane.de; 2 Department of Anesthesiology, University Hospital Ulm, 89081 Ulm, Germany

**Keywords:** midazolam, pharmacokinetics, pharmacodynamics, therapeutic drug monitoring, adverse drug reaction, personalized medicine, biomarkers, pharmacogenomics, drug therapy optimization

## Abstract

Midazolam, a short-acting benzodiazepine, is widely used to alleviate patient anxiety, enhance compliance, and aid in anesthesia. While its side effects are typically dose-dependent and manageable with vigilant perioperative monitoring, serious cardiorespiratory complications, including fatalities and permanent neurological impairment, have been documented. Prolonged exposure to benzodiazepines, such as midazolam, has been associated with neurological changes in infants. Despite attempts to employ therapeutic drug monitoring for optimal sedation dosing, its efficacy has been limited. Consequently, efforts are underway to identify alternative predictive markers to guide individualized dosing and mitigate adverse effects. Understanding these factors is crucial for determining midazolam’s suitability for future administration, particularly after a severe adverse reaction. This article aims to elucidate the factors influencing midazolam’s pharmacokinetics and pharmacodynamics, potentially leading to adverse events. Finally, a case study is presented to exemplify the complex investigation into the causative factors of midazolam-related adverse events.

## 1. Introduction

Midazolam, a fast- and short-acting benzodiazepine with an imidazole structure, has anxiolytic, muscle relaxant, anticonvulsant, sedative, hypnotic, and amnesic properties. There are different formulations of midazolam for different medical needs. In its intravenous injection form, midazolam serves as a sedative, anxiolytic, and amnesic agent administered before or during diagnostic, therapeutic, or endoscopic procedures, as well as prior to surgery. It may also be used to induce general anesthesia, as part of a balanced anesthesia approach, or as a continuous intravenous infusion for sedation in intubated and mechanically ventilated patients in critical care settings or for palliative sedation therapy. Oral formulations are utilized for preoperative sedation, anxiolysis, and amnesia, or before diagnostic, therapeutic, or endoscopic procedures in monitored environments. In cases of prolonged, acute, convulsive seizures, intra-muscular, buccal, or nasal formulations of midazolam are available for treatment. Midazolam onset of action is rapid regardless of the route, within 2 min (intravenous/intramuscular) to 15 min (oral). Midazolam exists in a pH equilibrium between closed and open benzodiazepine ring structures. The benzodiazepine ring of midazolam opens at a lower pH. At physiologic pH, the ring closes and the molecule becomes lipid soluble, allowing rapid penetration across the blood–brain barrier.

Dosage should be individualized, and dose titration typically involves administering bolus doses ranging from 0.1 to 0.3 mg/kg, along with a maintenance dose of 0.03–0.2 mg/kg per hour, as seen in ventilated patients, for instance. Midazolam is commonly administered intravenously in single doses ranging from 0.5 mg to 2.5 mg to achieve conscious sedation of patients undergoing endoscopy or minor surgery. A single 10 mg dose can be administered via intramuscular injection for the treatment of status epilepticus in adults.

Midazolam has a wide therapeutic range; however, toxicities can occur, particularly with higher doses and when administered intravenously. Midazolam toxicity manifests as central nervous system depression, ranging from drowsiness to coma. In mild to moderate cases, symptoms may include drowsiness, confusion, difficulty speaking, lethargy, a hypnotic state, reduced reflexes, lack of coordination, and decreased muscle tone. Occasionally, paradoxical or disinhibitory reactions may arise. In severe cases of overdose, patients may experience respiratory depression and coma. Overdosing on benzodiazepines, especially when combined with other central nervous system depressants like opioids, can be fatal. Various case reports detail adverse events, such as hypoxemia, inadequate sedation, and delayed recovery, associated with midazolam use.

The attempt to use therapeutic drug monitoring (TDM) to determine the optimal individual dose for adequate sedation with midazolam was unfortunately not successful. Efforts have been made to identify other predictive markers that can be used to determine the optimal dose for individual patients to avoid major side effects and toxicities.

Interindividual differences in pharmacokinetics, such as the clearance and half-life of midazolam, can be substantial and differ by a factor of ≥10 [1,2]. The kinetics of midazolam are highly dependent on hepatic cytochrome P450 3A (CYP3A) enzyme activity, as evidenced, for example, by a ≥10-fold increase in the area under the plasma drug concentration–time curve (AUC) of midazolam when potent CYP3A inhibitors are administered concomitantly. The AUC for the CYP3A4 substrate midazolam varied up to 400-fold in healthy volunteers exposed sequentially to the CYP3A4 inhibitor itraconazole and its transcriptional inducer rifampicin [3]. Midazolam is therefore recommended by the Food and Drug Administration (FDA) as a “clinical index substrate” for drug–drug interaction studies.

Factors such as co-administered drugs, age, sex, or inflammation have been shown to affect CYP3A activity and midazolam kinetics. Despite the influence of these non-genetic factors, genetic makeup could also provide an important contribution to the interindividual variability in the pharmacokinetics and dynamics of midazolam.

The heritability of midazolam plasma clearance was estimated to be 96% using a repeated drug administration approach [4]. In a study on the heritability of non-induced CYP3A activity conducted on twins, genetic effects accounted for 15% and 73% of the variation in the midazolam AUC and the 1′-hydroxy-midazolam (1-OH-M) AUC, respectively [5]. Although studies differ greatly in their estimates of the genetic contribution to CYP3A activity, they do not exclude a significant influence of genetics. Analogous to the important pharmacogene *CYP2D6*, for which genotyping to determine metabolizer status has become part of routine care, pharmacogenetic markers have also been sought for *CYP3A* genes. However, to date, no consistent association between *CYP3A4/5* genetic variation and pharmacokinetic/pharmacodynamic responses to midazolam that is useful for routine pharmacogenetic testing has been established [6,7,8]. Although many single nucleotide polymorphisms (SNPs) within the *CYP3A* locus have been identified, these variants appear to explain only a minority of the total heritability inferred indirectly from population data. Because of this “lack of heritability” problem, no clinical guideline has been established to guide midazolam prescription based on *CYP3A* genotype.

This review discusses the reasons why the TDM of midazolam is ineffective for dose individualization, for example, in long-term sedated patients, despite a clear medical need for a straightforward marker to adjust sedation depth and avoid toxicities, in light of potential drug–drug interactions and the effect of comorbidities of these often critically ill patients on pharmacokinetics. Furthermore, we describe several factors that contribute to the substantial interindividual variability in the pharmacokinetics and pharmacodynamics of midazolam. These factors include non-genetic variables such as sex, nutritional status, concomitant medication use, and the presence of comorbid conditions such as renal or hepatic dysfunction and inflammatory diseases. Genetic influences, including polymorphisms in genes encoding metabolizing enzymes or the GABA_A_ receptor, as well as epigenetic factors, are also discussed. Finally, a case is presented that illustrates the problem of “lack of heritability”.

## 2. TDM of Midazolam

TDM assists clinicians in determining optimal drug dosages for individualized treatment, particularly in cases where medications with narrow therapeutic indexes are prescribed for long-term therapy. Monitoring of analgosedation in continuous infusion therapy of midazolam, such as for analgosedation in mechanically ventilated patients, is crucial to avoid inadequate sedation and its complications. Suboptimal sedation levels can lead to various adverse effects, including hypercatabolism, immunosuppression, and unintended extubation, while excessive sedation may prolong mechanical ventilation and increase susceptibility to pneumonia and neuro-psychological impairment [9]. TDM of midazolam (as either a replacement or complement to clinical scores or neurophysiological monitoring) has been proposed to facilitate personalized treatment, ensure an appropriate depth of sedation, minimize toxicity, and address potential drug–drug interactions and comorbidities that affect drug metabolism and elimination [9]. However, TDM for midazolam is complex due to its active metabolite, 1-OH-M, necessitating the measurement of both drug and metabolite concentrations for a comprehensive assessment of effect. Clinically useful TDM requires a strong correlation between plasma concentration and clinical effect. While population-level data show a general correlation between plasma concentrations of midazolam and its active metabolite and sedative effect [10], significant individual variability complicates the prediction of sedation levels at specific midazolam concentrations in individual patients [9,10,11]. Generally, achieving deep sedation, defined as patients being asleep and reacting to pain induction, requires midazolam levels spanning a wide range of 100–2400 ng/mL in individual patients [12]. Therefore, midazolam does not meet a prerequisite for effective TDM, namely low interindividual pharmacokinetic and pharmacodynamic variability, and thus a clear concentration–response relationship. As a result, midazolam is not a drug for which TDM has demonstrated clinical value and is frequently monitored.

The challenge in correlating targeted plasma levels of midazolam with specific degrees of sedation is attributed to inter-subject variability in midazolam pharmacokinetics and pharmacodynamics, influenced by factors such as polypharmacy with drug interactions and complex metabolism, particularly in critically ill patients [9,10,11]. The subsequent section of the review explores factors influencing the pharmacokinetics and pharmacodynamics of midazolam, collectively contributing to inter-subject variability.

## 3. Transport and Metabolism of Midazolam

The ATP-binding cassette transporter P-glycoprotein (P-gp) is expressed on the apical membrane of mucosal cells in the intestine. Functioning as an ATP-dependent efflux pump with broad substrate specificity, P-gp facilitates the normal excretion of xenobiotics back into the gut lumen, consequently reducing the oral bioavailability of certain pharmaceutical drugs. The substrate specificities of CYP3A and P-gp are thought to overlap. Therefore, experimental studies have been performed to determine whether midazolam is a P-gp substrate, with conflicting results [13,14]. Takano et al. observed no inhibitory effect of the P-gp inhibitor verapamil on the basal-to-apical transport of midazolam in Caco-2 cells and concluded that P-gp is not involved in the transport of midazolam in the intestinal epithelia [13]. A more detailed investigation using a P-gp ATPase assay, efflux inhibition studies, and transport studies of midazolam across MDR1-MDCK and 1-α,25-dihydroxy vitamin D3-induced Caco-2 monolayers with and without the P-gp inhibitor GF120918 revealed that midazolam has the characteristics of a highly permeable P-gp substrate [14]. Substrates with high permeability, such as midazolam, exhibit rapid transmembrane movement, leading to significantly faster permeation into intestinal epithelial cells than P-gp efflux [14]. Therefore, the transport of highly permeable drugs remains largely unpolarized, suggesting that P-gp does not significantly impact their oral bioavailability [15]. Another class of transport proteins, known as membrane solute carrier transporters, typically plays a critical role in the cellular uptake of drugs. However, experiments conducted in *Xenopus laevis* oocytes or HEK293 cells expressing solute carriers revealed that midazolam did not function as a substrate for any of the tested transporters, including the organic anion transporters OAT1, OAT2, and OAT3, the organic cation transporters OCT1 and OCT2, and the organic-anion-transporting polypeptides Oatp1b2, OATP1A2, OATP1B1, OATP2B1, and OATP1B3 [16,17]. No study to date has investigated whether transport proteins are involved in the distribution or excretion of midazolam metabolites, some of which are also pharmacologically active [18].

Despite its rapid absorption following oral administration, the bioavailability of midazolam is relatively low at 44% due to a significant first-pass effect. Midazolam exhibits 94% plasma protein binding, and only 0.5% is excreted unchanged [19]. Because of extensive hepatic metabolism, hepatic impairment reduces the clearance of midazolam, leading to a subsequent increase in its terminal half-life. In a study comparing patients with liver cirrhosis to healthy controls, oral bioavailability significantly increased (76% vs. 38%), while midazolam elimination was notably delayed, demonstrated by lower total clearance (3.34 vs. 5.63 mL/min/kg), a lower total elimination rate constant (0.40 vs. 0.72 h-1), and a longer elimination half-life (7.4 vs. 3.8 h) [20]. Consequently, drug labels include a warning that clinical effects in patients with hepatic impairment may be more pronounced and prolonged, recommending dose reductions and the careful monitoring of vital signs in such patients.

**Figure 1:**
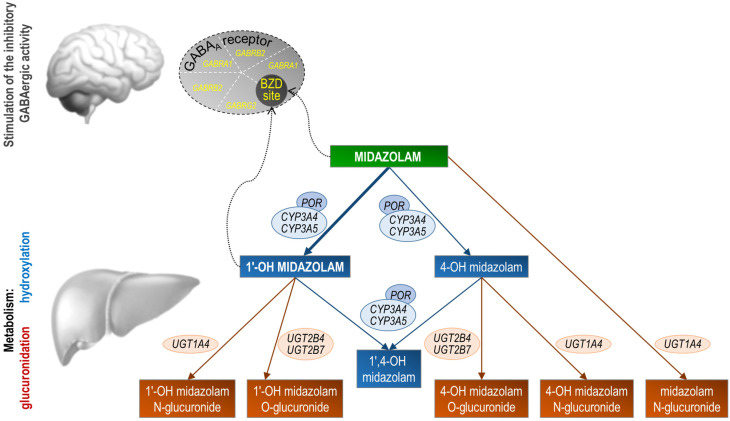
Stylized representation of midazolam pharmacokinetics and pharmacodynamics. Midazolam is primarily metabolized in the liver and gut by human cytochrome P450 3A (CYP3A4) enzymes to its pharmacologic active metabolite, 1′-OH midazolam (which accounts for approximately 70% of the biotransformation products), followed by glucuronidation of the 1′-hydroxyl metabolite. Studies in humans suggest that 1′-OH midazolam is as potent as the parent compound at the GABA_A_ receptor. Conversely, 4-OH-M’s low receptor affinity (7% compared to midazolam) and limited formation (less than 5% of midazolam’s biotransformation products) suggest it has negligible contribution to the overall activity of midazolam. Genes coding for the midazolam metabolic enzymes or the subunits that form the midazolam target receptor are shown. *POR*, cytochrome P450 oxidoreductase; *UGT*, UDP-glucuronosyltransferase; *GABRA1*, GABA_A_ receptor α1 subunit; *GABRB2*, GABA_A_ receptor β2 subunit; GABRG2, GABA_A_ receptor γ2 subunit; BZD site, benzodiazepine binding site at the GABA_A_ receptor.

The first step in the metabolism of midazolam is hydroxylation. The 1-OH-M metabolite comprises 60% to 70% of the biotransformation products of midazolam, while 4-hydroxy-midazolam (4-OH-M) constitutes 5% or less. Small amounts of a dihydroxy derivative, 1,4-OH-M, have also been detected. Hydroxylation is catalyzed by the enzymes of the CYP3A subfamily, including CYP3A5, CYP3A4, and CYP3A7 (Figure 1). However, CYP3A7 is only expressed in fetal tissue and therefore only plays a minor role later in life. Studies on drug–drug interactions involving potent CYP3A inhibitor drugs such as clarithromycin or ketoconazole underscore the significance of CYP3A enzymes in midazolam metabolism. A single dose of midazolam co-administered with oral clarithromycin (500 mg twice daily for 4 days) increased the midazolam AUC_0–∞_ by 219% after intravenous midazolam administration and 550% after oral midazolam administration [21]. Co-administration of a single dose of midazolam with oral ketoconazole (400 mg daily for 5 days) resulted in a 1296% increase in midazolam AUC_0–∞_ after the oral administration of midazolam [22].

The CYP3A4 and 3A5 enzymes, which share approximately 85% sequence homology, have overlapping substrate specificity. However, the affinity for CYP3A4 over CYP3A5 varies among CYP3A substrates. Using human liver microsomes, Tseng et al. showed in vitro that CYP3A5 contributes 55% and 44% to the 1′- and 4-hydroxylation of midazolam, respectively, suggesting that midazolam 1′-hydroxylation is more specific for CYP3A5 [23]. However, the in vitro data do not correspond to human in vivo data. In humans, there are individuals known as CYP3A5 expressors, who have functional CYP3A5 enzymes, and non-expressors, who lack functional CYP3A5 enzymes due to genetic variations. CYP3A5 non-expressors compared to expressors have been used to estimate the relative contribution of CYP3A4 and CYP3A5 for midazolam clearance. Among healthy volunteers, the pharmacokinetics of intravenously administered midazolam did not differ between CYP3A5 expressors and non-expressors, suggesting that hepatic CYP3A5 generally plays a minor role in midazolam metabolism [24]. However, the inhibition of CYP3A4 resulted in decreased midazolam clearance, specifically in CYP3A5 non-expressors compared to CYP3A4 expressors [24], indicating that CYP3A5 serves as an alternative metabolic pathway for midazolam, particularly when CYP3A4 is not functional.

1-OH-M is the major metabolite of midazolam and has been shown to account for 95% of the net intrinsic clearance of midazolam in human liver microsomes [25]. After hydroxylation, midazolam is further metabolized in the second step by the UDP-glucuronosyltransferases (UGTs) 1A4, 2B4, and 2B7, producing 1′-hydroxy-midazolam-glucuronide (1-OH-MG) as the main metabolite [26]. The amount of 1-OH-M excreted as a conjugate in the urine of healthy volunteers exceeded 75% of the initially administered dose compared to 4% for the minor primary metabolite 4-OH-M and 6% for the second minor secondary metabolite 1′,4-dihydroxy-midazolam. Approximately 1–2% is also excreted directly as glucuronylated midazolam generated by UGT1A4 [27,28].

## 4. Pharmacokinetics and Pharmacodynamics of Midazolam Metabolites

Several studies have explored the pharmacological activities of certain midazolam metabolites. The primary metabolite, 1-OH-M, has been shown to exhibit similar acute pharmacodynamic effects as midazolam in studies involving healthy volunteers [29,30], thus contributing to midazolam’s overall pharmacological activity. In a randomized, double-blind, crossover trial, Mandema et al. evaluated the pharmacodynamic response using saccadic eye movement and electroencephalographic (EEG) measurements in eight healthy volunteers following the intravenous administration of midazolam or 1-OH-M. Concentration–effect relationships of midazolam and its main metabolite, 1-OH-M, were found to be comparable [30]. Consistent with these findings, in vitro binding studies demonstrated that the affinity of 1-OH-M for the cerebral benzodiazepine receptor (with an affinity constant of 2.2 nmol/L) closely resembled that of midazolam (1.4 nmol/L) [31]. However, the elimination half-life of the metabolite 1-OH-M is shorter than that of midazolam, approximately 1 h versus 1.5 to 3.0 h [32,33]. This faster inactivation of 1-OH-M results in a quicker decrease in its pharmacodynamic effect compared to midazolam. This aligns with the findings of Johnson et al., who conducted a pharmacokinetic–pharmacodynamic analysis of single-dose oral midazolam and concluded that the metabolite 1-OH-M contributed approximately half to the sedative effect compared to the parent drug [34]. The analysis was based on study data on the pharmacokinetics of midazolam and its active 1-OH metabolite and their contribution to the sedative effect in 45 children undergoing day surgery [34].

The affinity of 4-OH-M for the benzodiazepine receptor was found to be only 7% compared to midazolam. Therefore, considering that 4-OH-M accounts for 5% or less of the biotransformation products of midazolam, this metabolite is unlikely to substantially contribute to the overall pharmacologic activity of midazolam. The binding affinity of 1-OH-MG to the cerebral benzodiazepine receptor was only about 10 times weaker than that of midazolam; therefore, this glucuronidated metabolite may also contribute to the overall pharmacological activity, especially if it accumulates [31].

Franken et al. demonstrated that the clearance of 1-OH-MG correlates with the estimated glomerular filtration rate, which serves as an indicator of renal function, suggesting that 1-OH-MG is mainly excreted via the kidneys [35]. Serum concentration monitoring in patients with severe renal failure showed high concentrations of 1-OH-MG, whereas the concentrations of the unconjugated metabolite and the parent drug were below the sedative range or even below the detection range. Moreover, the accumulation of 1-OH-MG has been associated with the prolonged sedative effects frequently seen in critically ill patients with renal impairment [31]. Accordingly, the drug labels include a warning that in patients with renal impairment, the excretion of midazolam and its metabolites may be slowed, which may result in prolonged sedation possibly including clinically relevant respiratory and cardiovascular depression, but they do not provide specific dose adjustment recommendations.

## 5. Effect of Sex and Nutritional Status on the Pharmacokinetics of Midazolam

Sex influences numerous pharmacokinetically important parameters, including the expression of drug-metabolizing enzymes and transporters [36,37]. Analyses of CYP3A4 in the human liver have shown that relative mRNA expression was significantly higher in females than in males, by approximately 26% [38], and that CYP3A4 protein levels are approximately 2-fold higher in female than in male liver tissue [38,39,40,41]. Men appear to have higher activity than women for some CYP isoenzymes such as CYP3A5, for the drug efflux transporter P-gp, and for some UGT isoforms such as UGT2B4 [42,43]. Many clinical studies have indicated that women metabolize CYP3A4 substrates such as midazolam more rapidly than men [44]. In a phase I clinical trial, Tsunoda et al. observed that female subjects exhibited a mean midazolam clearance 1.6-fold higher than male subjects when adjusted for weight [45]. Notably, this difference disappeared in the presence of the CYP3A inhibitor ketoconazole, confirming a contributory role of sex differences in CYP3A activity to the observed variations in midazolam kinetics [45]. One endogenous factor that regulates the sexually dimorphic expression of hepatic CYP3A4 is growth hormone (GH), which exhibits a sexually dimorphic pattern of secretion. The masculine GH profile is episodic, while the feminine profile is continuous. Masculine-like episodic GH profiles suppress CYP3A4 expression, whereas feminine-like continuous GH profiles induce it [46,47].

In a meta-analysis of ten studies involving 409 healthy volunteers, women showed a 16% higher weight-corrected midazolam oral clearance and 20% higher systemic clearance than men, although no significant difference in the AUC after the oral dosing of midazolam was noted between the sexes [48]. Population pharmacokinetic modeling, utilizing data from 13 studies including 138 healthy volunteers, indicated that women displayed an 11% higher mean weight-corrected total body midazolam clearance and a 28% higher oral clearance compared to men [49]. Despite the significantly greater midazolam clearance in women, the overall magnitude of observed disparity, however, may have negligible clinical implications.

Nutritional status is another factor that potentially influences CYP3A4 activity and thus midazolam metabolism [50]. In a randomized, controlled, cross-over study, 36 h fasting altered midazolam metabolism in different ways. Here, CYP3A4-mediated midazolam metabolism increased by 12%, while UGT-mediated metabolism decreased by 13% [51]. The findings indicate that short-term fasting diminishes the activity of UGT phase II drug-metabolizing enzymes, reflected by reduced UGT-mediated midazolam metabolism. Conversely, it augments the activity of the phase I drug metabolizing enzyme CYP3A4, as evidenced by the increased CYP3A4-mediated metabolism of midazolam. The human studies align with experimental findings showing that short-term fasting significantly increases mRNA expression of the orthologues of human *CYP3A4* in both rats and mice [52,53]. A high-fat diet did not affect the systemic clearance of midazolam or its metabolites in a study with healthy volunteers [54]. In terminally ill patients receiving midazolam, hypalbuminemia, possibly due to a catabolic state, was significantly associated with decreased midazolam clearance [35].

Obesity is a factor that may influence the pharmacokinetics of midazolam, as demonstrated in a study comparing normal weight (*n* = 12) and obese patients (*n* = 20, body mass index 40–68 kg/m^2^) [55]. While the clearance of midazolam did not show a significant difference between the groups, the mean half-life was notably greater in the obese group (5.9 versus 2.3 h). This was attributed to an approximately 50% increase in the volume of distribution corrected for total body weight [55].

## 6. Impact of Inflammation on the Pharmacokinetics of Midazolam

During acute or chronic inflammation, released proinflammatory cytokines such as interleukin (IL)-1β, tumor necrosis factor (TNF)-α, and IL-6 act as signaling molecules to elicit marked changes in liver gene expression profiles and lead to the strong downregulation, and activity reduction, of many drug-metabolizing enzymes, including CYP3A4 [56,57,58,59]. A negative correlation between plasma TNF-α concentrations and midazolam clearance was found in patients with chronic inflammation [60], and midazolam clearance correlated with plasma levels of the inflammatory marker C-reactive protein in critically ill patients with acute inflammation [61]. In patients with severe inflammatory syndrome due to acute respiratory syndrome-coronavirus 2, C-reactive protein elevation was significantly associated with a decrease in the 1-OH-M/midazolam plasma ratio, suggesting reduced metabolism of midazolam by CYP3A [62].

The mechanism behind these effects is at least partly attributed to the transcriptional repression of CYP3A4 [57,58,63]. For instance, the treatment of human hepatocytes with IL-6 in vitro resulted in a decreased expression of CYP3A4 at both mRNA and protein levels, along with reduced enzymatic activity [64]. Experimental studies suggest that IL-6 down-regulates CYP3A4 through translational induction of the CCAAT/enhancer binding protein (C/EBP) β-LIP isoform, which competes with and antagonizes constitutive C/EBP transactivators [63]. Protein expression of the transcription factor C/EBPβ occurs by alternative in-frame translation initiation at consecutive start sites from a single transcript to generate three isoforms, two long and one truncated liver inhibitory protein (LIP). The long C/EBPβ isoforms are considered gene activators, whereas the LIP isoform reportedly acts as a dominant-negative repressor [63].

The inflammatory cytokines IL-1β and TNF-α or bacterial lipopolysaccharides activate nuclear factor kappa B (NF-κB), which can strongly inhibit human CYP3A4 expression [65]. Gene silencing experiments in primary human hepatocytes indicate that retinoid X receptor α (RXRα) plays a central role in mediating transcriptional downregulation [56,66]. During inflammation, endotoxin prompts the rapid loss of nuclear-localized RXRα [66]. RXRα serves as a necessary heterodimerization partner for several key ligand-activated DNA-binding transcription factors, including pregnane X receptor (PXR), which regulates the expression of its target gene, *CYP3A4*, by binding to the gene’s promoter [56]. Experimental studies showed that the p65 subunit of NF-κB directly interacts with the DNA-binding domain of RXRα and prevents its binding to the consensus DNA sequences, thus inhibiting transactivation by the PXR/RXRα complex [65].

## 7. Genetic Factors—*CYP3A* Polymorphisms

The substrate specificities of CYP3A4 and CYP3A5 generally overlap, but the relative contribution of the metabolic capability of CYP3A5 compared with that of CYP3A4 is either reduced or, at best, equal depending on the substrate. CYP3A4 is the major contributor to midazolam metabolism, and CYP3A5 becomes relevant only when CYP3A4 activity is impaired [24]. Studies in human liver microsomes have shown that both CYP3A4 and CYP3A5 contribute to the metabolism of midazolam, but biotransformation with CYP3A5 is metabolically less active than that with CYP3A4 [67].

*CYP3A5* expression is polymorphic, with 25 allelic variants of *CYP3A5* listed on the Pharmacogene Variation Consortium website [68,69]. The most common nonfunctional variant, CYP3A5*3 (rs776746), results from a change from A to G at position 6986 of the gene, creating a cryptic splice site in intron 3. This alteration leads to abnormal mRNA splicing, generating an alternatively spliced isoform with an insertion from intron 3. Consequently, a premature termination codon occurs, rendering the protein nonfunctional [69]. Individuals with the CYP3A5*3/*3 genotype are considered non-expressors. The frequency of this genotype varies significantly among populations. In White Europeans, the allele frequency of CYP3A5*3 is approximately 0.94, with around 89% of the population carrying two 3 alleles and thus being non-expressors. In contrast, only 42% of Asians and 29% of Africans are non-expressors, i.e., carry the CYP3A5*3/*3 genotype. There is no difference in 1-OH-M formation between CYP3A5 expressers and non-expressers, suggesting that the 1′-OH hydroxylation of midazolam is primarily catalyzed by CYP3A4 [70,71,72]. Moreover, the pharmacodynamics of midazolam are not affected by the CYP3A5*3 variant. This was demonstrated in a prospective study involving 100 Korean patients undergoing upper gastrointestinal endoscopy under sedation with midazolam, where the sedation grade showed no association with the CYP3A5*3 genotype [71]. Similarly, in a cohort of 71 Caucasian patients receiving prolonged sedation during intensive care treatment, no link between the CYP3A5*3 genotype and sedation depth, as assessed by the Ramsay score, was identified [72].

Two other commonly studied *CYP3A5* loss-of-function alleles, *6 (rs10264272) and *7 (rs41303343), result in nonfunctional proteins due to aberrant splicing, with the absence of exon 7 and a frameshift leading to premature termination, respectively. These alleles are relatively common in African populations (0.12–0.15) but are rare or absent in White and Asian populations (0–0.003) [73].

CYP3A4 activity can vary significantly among individuals, by up to a factor of 100 [74]. However, unlike the distribution of CYP2D6 activity in the population, which exhibits multiple peaks allowing for clear differentiation between extensive and poor or rapid metabolizers, the distribution of CYP3A4 activity in the population is continuous and unimodal [75,76]. This finding suggests that the regulation of CYP3A4 activity involves multiple genes, with individual genetic factors playing a minor role [75]. As a result, current FDA drug labeling does not provide information on genomic biomarkers of *CYP3A4* [77].

To date, approximately 9800 SNPs have been identified in the *CYP3A4* gene (dbSNP database), with 35 core alleles currently listed by PharmVar [78]. The functional implications of some of these core alleles are not yet clear. Using the allele definitions issued by PharmVar, the Royal Dutch Association for the Advancement of Pharmacy—Pharmacogenetics Working Group listed the following allele function assignments and phenotype mapping results: normal function of *1 suballeles including *1A, *1B, and *1G; decreased function of alleles *8, *11, *12, *13, *16, *17, *18, and *22; complete loss of function of alleles *6, *20, and *26 [2,76,79].

The extensively studied subvariant CYP3A4*1B appears to have no or very limited functional impact on CYP3A4 [80], contrary to some initial reports influenced by its linkage disequilibrium with CYP3A5*1. These early reports suggest that the CYP3A4*1B allele (defined by the promoter SNP rs2740574, −392A>G) exhibits greater enzymatic activity than the wild-type allele, CYP3A4*1A, resulting in an increased systemic clearance of midazolam [81]. However, in approximately 80% of Caucasians carrying CYP3A4*1B, this allele is in linkage disequilibrium with the fully active *1 allele of *CYP3A5*. Therefore, the clinical phenotype initially associated with CYP3A4*1B is likely attributable to CYP3A5 activity [82].

The CYP3A4*2 allele, characterized by the c.664T>C SNP, results in a serine-to-proline amino acid residue exchange at codon 222, potentially altering the three-dimensional structure of CYP3A4 because the proline residue is a known helix breaker [83]. Two in vitro studies have linked this allele with a marked functional defect in CYP3A4 activity [84,85], demonstrating reduced activity toward midazolam in vitro, although its clinical relevance remains undemonstrated.

For CYP3A4*3, an allelic frequency of approximately 1% has been reported, but unlike CYP3A4*2, no correlation with enzyme activity has been identified [83,86,87]. There have been no clinically significant associations reported for CYP3A4*3, raising doubts about its functional role, particularly when compared to CYP3A4*2.

The CYP3A4*4, CYP3A4*5, and CYP3A4*6 alleles were initially discovered in 102 Chinese subjects, with allelic frequencies of 1.5%, 0.98%, and 0.5%, respectively, all linked to reduced CYP3A4 activity as indicated by the urinary 6β-hydroxycortisol-to-free cortisol ratio [88]. Like Hsieh et al.’s study [88], Wang et al. found that the CYP3A4*4 allele was associated with decreased CYP3A4 activity, resulting in an enhanced lipid-lowering effect of the CYP3A4 substrate simvastatin [89]. To date, the CYP3A4*4 allele has only been identified in the Chinese population [2,90,91]. The CYP3A4*6 allele (rs4646438, insertion c.830dupT) causes a frameshift and loss of enzymatic activity and is found in all populations, albeit with a very low allele frequency.

CYP3A4*7 (p.Gly56Asp), CYP3A4*8 (p.Arg130Gln), CYP3A4*9 (p.Val170Ile), CYP3A4*10 (p.Asp174Asn), CYP3A4*11 (p.Thr363Lys), CYP3A4*12 (p.Leu373Phe), CYP3A4*13 (p.Pro416Arg), and CYP3A4*20 (a frameshift variant with a premature stop codon) have been identified in Caucasian DNA samples. However, while some of these alleles have been associated with altered CYP3A4 activity in vitro, their reported allelic frequencies are very low [2,86,87,92]. Other coding SNPs such as CYP3A4*14 (p.Leu15Pro), CYP3A4*15 (p.Arg162Gln), CYP3A4*16 (p.Thr185Ser), CYP3A4*19 (p.Pro467Ser), and CYP3A4*21 (p.Tyr319Cys) are rare, particularly in Caucasians [2,86,93].

The CYP3A4*17 (p.Phe189Ser) allele is linked to reduced clearance of certain substrates, including midazolam, testosterone, and nifedipine, compared to the *CYP3A4* wild-type allele [83,94]. The two total loss-of-function variants CYP3A4*20 (frameshift variant, c.1461dup) and CYP3A4*26 (premature stop codon, p.Arg268Ter) were initially identified due to anomalous pharmacokinetics in certain individuals [92,95]. These genetic variants have a very low frequency in the global population (less than 0.01%), but their prevalence varies across different subpopulations. For instance, CYP3A4*20 is present in the Spanish population with an allele frequency as high as 1.2% [96].

The decreased-function CYP3A4*22 allele was first reported in 2011 [97] and is characterized by a C>T substitution at position 15,389 (rs35599367) in intron 6 of *CYP3A4*, resulting in alternative RNA splicing [97]. The effect of the CYP3A4*22 allele on hepatic expression was confirmed by measuring total *CYP3A4* mRNA levels in liver samples. Livers with the wild-type genotype, CYP3A4*1/*1 (CC), exhibited 1.7-fold higher mRNA levels than those from carriers of the CYP3A4*22 (CT or TT) variant allele. The CYP3A4*22 allele accounted for 7% of the variability in *CYP3A4* mRNA expression. In additional microsomal liver samples, CYP3A4 activity, as assessed by the testosterone 6β-hydroxylation rate, was 2.4-fold higher in individuals with CYP3A4*1/*1 than in heterozygous CYP3A4*22 samples. The authors concluded that the presence of the CYP3A4*22 allele reduces both CYP3A4 liver expression levels and enzyme activity [97]. The SNP explained up to 12% of the variability observed in CYP3A4 activity. The association of CYP3A4*22 with reduced metabolic activity has been demonstrated for several CYP3A4 substrates, including midazolam [70,76]. Midazolam levels increased by 32% in individuals carrying the CYP3A4*22 variant compared to those in patients with the homozygous wild-type genotype [70]. This genotype-specific difference in CYP3A4 activity also affects metabolite formation. Plasma concentrations of the metabolite 1-OH-M and the corresponding metabolic ratio (the ratio of the metabolite to unchanged midazolam) were 19.8% and 21.8% lower, respectively, in carriers of the CYP3A4*22 variant compared to wild-type patients [70]. Overall, the intronic CYP3A4*22 variant appears to be the most relevant single *CYP3A4* variant influencing CYP3A4 activity, with a minor allele frequency of 5% in Europeans and approximately 3% in admixed Americans, but it is rare in many other populations [98]. Accordingly, the Association for Molecular Pathology Clinical Practice Committee’s Pharmacogenomics Working Group currently recommends the inclusion of CYP3A4*22 together with CYP3A4*20, CYP3A5*3, *6, and *7 alleles as a panel of variant alleles in clinical pharmacogenetic genotyping assays for CYP3A substrate drugs [99]. While the impact of certain *CYP3A4* genetic polymorphisms on midazolam pharmacokinetics, notably the effect of CYP3A4*22 on the 1-OH-M/midazolam metabolic ratio, has been demonstrated, their effect on pharmacodynamics, such as the depth of sedation, has not yet been investigated and requires further establishment.

Although many SNPs within the *CYP3A* locus have been identified, these variants appear to explain only a minority of the phenotypic variability. This was demonstrated in a study involving 21 healthy subjects genotyped for a broad panel of *CYP3A4* and *CYP3A5* alleles, where CYP3A activity was measured using midazolam as a probe substrate [100]. Importantly, the *CYP3A* genotypes did not adequately account for the interindividual variability in the activity of these two isozymes, highlighting the issue of “missing heritability” of CYP3A [100].

## 8. Transcriptional Regulation of *CYP3A* Genes

Significant (~50-fold) interindividual differences in CYP3A4 expression levels are observed in the human liver. There is growing consensus that this variability cannot be solely attributed to genetic polymorphisms in *CYP3A4* but is instead influenced by polymorphisms/variability in CYP3A4 regulators [8,101,102]. The expression of CYP3A enzymes is known to be controlled by numerous signaling pathways and transcription factors. Constitutive transcriptional regulation involves transcription factors such as CCAAT/enhancer-binding protein α and β (C/EBPα and C/EBPβ) [63,103,104], hepatocyte nuclear factors (HNFs), particularly HNF1α, HNF3γ [103], and HNF4α [105,106,107], upstream stimulatory factors (USFs) [108], and estrogen receptor alpha (ESR1) [109]. ESR1 has been shown to account for 63% and 42% of the variability in the constitutive expression of CYP3A4 and CYP3A5 in human liver samples, respectively, indicating its significant role as a regulator of CYP3A expression [110].

CYP3A4 induction by endo- and xenobiotics primarily relies on the pregnane X receptor (PXR, alternate names: SXR, PAR, and *NR1I2*), and, to a lesser extent, the constitutive androstane receptor (CAR, *NR1I3*). Upon binding to its ligand (which can be a drug or xenobiotic), PXR or CAR undergoes a conformational change enabling it to form a heterodimer with the RXR. This dimeric complex, upon translocation from the cytoplasm into the nucleus, binds to responsive elements in the *CYP3A4* promoter region, thereby regulating its transcription. These responsive elements, including the proximal PXR responsive element, the distal xenobiotic-responsive enhancer module (XREM), and the far distal enhancer module (FDEM), consist of various repeats of the consensus motif, AG(G/T)TCA [107,111,112,113]. The repeats can be arranged in different configurations such as direct repeats (DR) separated by 3–5 nucleotides (DR3, DR4, or DR5) or everted repeats (ER) separated by 6 or 8 nucleotides (ER6 or ER8). The proximal PXR responsive element contains an ER6 [114], while the distal XREM contains a DR3 [115] and the FDEM contains an ER6 motif [116].

PXR, predominantly expressed in the liver and small intestine, serves as a crucial xenosensor, playing a significant role in the pharmacokinetics of CYP3A4 substrate drugs such as midazolam. For instance, in healthy volunteers exposed to the known PXR agonist and CYP3A4 transcriptional inducer rifampicin, the AUC for the CYP3A4 substrate midazolam decreased to 2.3% of the AUC of unexposed individuals [3].

Genetic variants within transcriptional regulator genes also play a role in modulating CYP3A expression levels. Lamba et al. screened potential regulatory regions in the PXR gene *NR1I2* for sequence variations that could explain the observed variability in the hepatic expression of PXR and its target *CYP3A4* [117]. *NR1I2* SNPs were genotyped in human liver donors characterized for *CYP3A4* mRNA expression and in primary human hepatocytes assessed for both basal and rifampin-inducible CYP3A4 activity. Among the identified SNPs, those consistently associated with phenotypic measures of *CYP3A4* included a promoter SNP 44477T>C (−1359), SNP 63396C>T within intron 1, and SNPs 56348C>A, 69789A>G, and 66034T>C [117]. Liver donors harboring variant *NR1I2* alleles exhibited altered hepatic expression of the PXR-regulated gene, *CYP3A4*, in comparison to those with wild-type *NR1I2* alleles. However, the impact of these polymorphisms on interindividual pharmacokinetic variations of midazolam remains unexplored.

The influence of the ligand-activated transcription factors PXR and CAR extends beyond CYP3A4, as they serve as transcriptional activators for at least 40 genes involved in drug transport and phase I/II metabolism [94]. These factors have been implicated in the transcriptional activation of genes crucial to the pharmacokinetics of midazolam, including cytochrome P450 oxidoreductase (*POR*) [118], *CYP3A4* [119], *CYP3A5* [120], *UGT1A4* [121], and *UGT2B7* [122]. PXR is activated by a wide range of xenobiotics, including antibiotics, antimycotics, herbal components [123], and benzodiazepines such as medazepam and midazolam [124]. PXR activation has been shown to induce not only the midazolam-metabolizing phase I enzyme CYP3A4 but also the phase II enzymes UGT1A4 and UGT2B7. While most benzodiazepines do not affect PXRs, midazolam exhibits slight PXR activation and weak induction of *CYP3A4* mRNA in human hepatocytes [124].

Other ligand-dependent transcriptional regulators of *CYP3A4* include the bile acid receptor FXR [125], the glucocorticoid receptor [126], the liver X receptors (LXRs) [127], and the vitamin D receptor (VDR) [128]. Several studies have suggested that peroxisome proliferator-activated receptor alpha (PPARα) also contributes to the constitutive and inducible regulation of CYP3A4. PPARα SNPs have been shown to influence hepatic CYP3A4 phenotypes [129,130]. Additional experiments demonstrated approximately a 3-fold induction of CYP3A4 in human hepatocytes by a potent PPARα agonist, while repression occurred with a PPARα antagonist and short hairpin RNA (shRNA)-mediated PPARα knockdown. A transcriptional profiling study [131] also underscored the impact of the lipid homeostasis regulator PPARα on CYP3A4.

In addition, NF-κB activation by inflammatory cytokines can potently suppress human *CYP3A4* expression [65], and, conversely, PXR activation suppresses inflammation through interaction with the NF-κB pathway [132]. The cytokine-mediated downregulation of *CYP3A4* during inflammation via the JAK/STAT pathway is clinically significant [63]. This process is particularly relevant in cancer patients because tumors can serve as sources of cytokines, leading to the substantial downregulation of CYP3A4 and other drug-metabolizing enzymes and transporters [58,133].

## 9. Regulation of CYP3A4 by Short Non-Coding Regulatory MicroRNAs

MicroRNAs (miRNAs) are approximately 22-nucleotide-long RNA molecules encoded by the genome, playing pivotal roles as gene expression regulators in eukaryotes. They exert post-transcriptional control by binding to target mRNAs, thereby modulating their translation or stability. Through targeting multiple mRNAs, individual miRNAs possess the ability to finely adjust or regulate gene expression within specific pathways. This regulatory versatility of miRNAs is particularly significant in xenobiotic metabolism, where the dynamic adaptation to environmental cues is crucial for metabolizing and eliminating substances. Indeed, studies have demonstrated the involvement of miRNAs in modulating the expression of genes involved in drug metabolism [119,120]. For example, *CYP3A4* was shown to be directly regulated by the miRNA miR-27b [134] and lower miR-27b levels were associated with higher CYP3A activity [135]. Nuclear receptors also serve as targets for miRNAs, as evidenced by the control of PXR by miR-148a, which influences CYP3A4 and CYP2B6 expression levels and the metabolism of xenobiotic drug substrates [136]. Additionally, HNF4α is regulated by miR-24 and miR-34a, with the overexpression of these miRNAs leading to reduced HNF4α levels and the downregulation of its target genes [137]. The vitamin D receptor (VDR), another transcriptional regulator of *CYP3A4*, is subject to regulation by the *CYP3A4*-targeting miR-27b, thereby establishing both indirect and direct mechanisms for the miRNA regulation of *CYP3A4* [134]. The same miRNA, miR-27b, also modulates PPARγ [138,139], while LXR has been shown to be regulated by miR-613 [140], further supporting the important role of miRNAs in hepatic gene regulation. Of particular interest for pharmacogenetic considerations are SNPs in miRNAs and miRNA binding sites, as well as miRNA copy number variations, which could impact their expression and function, thereby influencing target gene expression [141,142].

## 10. Genetic Factors—*UGT1A* Polymorphisms

The variability in midazolam clearance among subjects may not solely stem from variations in *CYP3A4* but also from differences in N-glucuronidation capacity. UGT1A4 is primarily expressed in the liver and participates in the phase II metabolism of midazolam [27,28]. Pharmacokinetic modeling has indicated that UGT1A4 plays a role in metabolism, especially in scenarios where CYP3A is inhibited, like CYP3A5 deficiency or the inhibition of CYP3A4 by interacting drugs [27]. Consequently, direct N-glucuronidation of midazolam might partly counterbalance the decrease in metabolic clearance caused by impaired CYP3A4 and/or CYP3A5 function [27,28].

Variability in the expression or activity of UGT1A4 could result in differences in drug response and toxicity among individuals [143]. Aueviriyavit et al. observed 2.5-fold interindividual differences in the expression levels of *UGT1A4* mRNA in human livers, attributed in part to genetic polymorphisms [144]. Currently, the Pharmacogenomics Laboratory (Université Laval) website lists eight core alleles, *1 to *8, for *UGT1A4* [145]. Among the extensively studied alleles are UGT1A4*2, characterized by a proline-to-threonine amino acid change at codon 24 due to a single C>A substitution, and UGT1A4*3, which leads to a leucine-to-valine amino acid change at codon 48 caused by a single T>G substitution [146]. These two allele-defining variants are located in the first exon of the *UGT1A4* gene and could alter enzyme activity, with UGT1A4*3 potentially increasing and UGT1A4*2 decreasing glucuronidation.

In vitro studies examining the effect of UGT1A4*2 on the metabolism of UGT1A4 substrates, such as olanzapine, clozapine, and lamotrigine, suggested that UGT1A4*2 might reduce enzyme activity [147,148,149]. Clinical investigations into the effects of UGT1A4*2 on the metabolism of UGT1A4 substrates have yielded less definitive results, showing, at most, a trend toward higher serum concentrations of the substrate lamotrigine [150]. Conversely, clinical studies have found that the UGT1A4*3 variant is linked to increased enzyme activity, resulting in decreased serum concentrations or an elevation in the glucuronidation rate of substrate drugs [150,151,152,153]. To date, no clinical study has explored the impact of UGT1A4*2 or UGT1A4*3 polymorphisms on midazolam or 1-OH-M metabolism.

## 11. Genetic Factors—Polymorphisms in GABA_A_ Receptor Genes

Benzodiazepines primarily exert their pharmacodynamic effects by augmenting the activity of the inhibitory neurotransmitter gamma-aminobutyric acid (GABA) in the central nervous system. This enhancement results in increased GABAergic neurotransmission, leading to sedative, anxiolytic, muscle relaxant, and anticonvulsant effects. Benzodiazepines bind to specific sites on GABA_A_ receptors, which are ligand-gated ion channels, thereby increasing the frequency of chloride channel opening. This hyperpolarizes neurons and suppresses neuronal excitability. GABA_A_ receptors in the brain generally consist of two α, two β, and one γ subunit. While the GABA binding site is located at the α-subunits, precisely at the contact site with the β-regions, benzodiazepines bind at the contact site of the α-subunit with the γ-subunit. However, not every α-subunit can form a binding site for benzodiazepines with the γ-subunit; only α1, α2, α3, and α5 can do so. Approximately 60% of GABA_A_ receptors have the α1β2γ2 configuration, while the combination α2β3γ2 accounts for approximately 15–20%, and the α3βnγ2 complex occurs in approximately 10–15%. Together, these three combinations make up 90% of all GABA_A_ receptors in the CNS. Limited knowledge exists regarding the potential influence of polymorphisms in the GABA_A_ receptor-encoding genes on the pharmacodynamics of midazolam. A study in an Asian population found that the intronic polymorphism rs4263535 in the GABA_A_ receptor subunit α1 gene (*GABRA1*) was significantly associated with deeper sedation induced by intravenous midazolam [154]. However, this observation has not yet been validated in an independent cohort, and the underlying biological mechanism responsible for the genotype–phenotype association of this noncoding intronic variant has not been investigated. Consequently, the effect of the rs4263535 polymorphism or other polymorphisms in linkage disequilibrium on the expression or function of the GABA_A_ receptor α1 subunit remains uncertain.

## 12. Epigenetics

Of particular interest is the epigenetic regulation of the important transcription factor and xenosensor PXR [136], whose functions are regulated at three epigenetic levels: by noncoding miRNAs, as previously described, through chromatin modifications, and via DNA methylation. Chromatin modifications are carried out, in part, through interaction with coregulator complexes, which include steroid receptor coactivators, corepressors (NcoR/SMRT), HNF4α, PPARγ coactivator 1α, and protein arginine methyltransferase 1. PXR can undergo post-translational modifications such as acetylation, phosphorylation, and sumoylation, while its promoter region can be subject to methylation at the CpG island. These factors collectively determine PXR activity, thereby affecting the magnitude and duration of PXR-regulated drug metabolic responses [155].

## 13. Case Example

The following case report of prolonged midazolam-associated sedation is intended to illustrate the clinical and pharmacogenetic factors that should be considered in the work-up. A Caucasian normal weight male child aged 4 years and 7 months underwent elective adenotonsillectomy and was sedated preanesthetically with midazolam followed by a standard anesthesia protocol. Midazolam syrup (0.65 mg/kg) was administered orally 40 min prior to surgery. Anesthesia was induced with 3.3 mg/kg of propofol before fentanyl administration (3.3 μg/kg). Muscle relaxation was achieved with mivacurium (0.2 mg/kg). General anesthesia was accomplished with desflurane supplemented with remifentanil (0.1 μg/kg/min). Additionally, 0.1 mg/kg ondansetron/0.15 mg/kg dexamethasone was used for the prophylaxis of postoperative nausea and vomiting, and 14 mg/kg dipyrone was used for postoperative pain management. The patient showed delayed recovery and prolonged respiratory depression for more than 5 h. Respiratory depression and sedation were reversed with 0.01 mg/kg flumazenil, suggesting that the delayed postanesthetic recovery was due to the effects of midazolam.

Delayed recovery from sedation is occasionally observed in critically ill patients due to liver failure and a decrease in the hepatic clearance of midazolam. Furthermore, it is well documented that the clearance of midazolam can be reduced by interaction with CYP3A-inhibiting drugs or food components (e.g., macrolide antibiotics, azole antimycotics, or grapefruit), resulting in prolonged sedation [156,157]. Our patient did not fall into either of the two categories described above, which poses challenges for differential diagnosis in such a rare case.

Despite the influence of environmental or clinical factors, genetics may influence the pharmacokinetics and pharmacodynamics of midazolam, as discussed above. In fact, a high degree of heritability was observed for hepatic CYP3A4 activity, as measured by midazolam plasma clearance [4]. We examined the patient’s germline DNA using whole-exome and targeted Sanger sequencing for the presence of functional SNPs or Indel variants that could explain the altered midazolam pharmacokinetics or pharmacodynamics. Candidate genes that may affect the pharmacokinetics or pharmacodynamics of midazolam include *CYP3A*, *POR* (the gene product, cytochrome P450 oxidoreductase, is a flavoprotein that donates electrons to microsomal P450 enzymes), *UGTs*, and genes encoding the five subunits of the GABA_A_ receptor, the molecular target of midazolam [158] (Figure 1).

The patient was identified as a homozygous carrier of the CYP3A5*3 allele, i.e., as a CYP3A5 non-expressor. Therefore, the phase I metabolism of midazolam in our patient, as in 90–95% of all Europeans, depends on CYP3A4 activity [159]. Our patient, however, did not carry any of the previously described *CYP3A4* decrease or loss-of-function alleles (e.g., *8, *11, *12, *13, *16, *17, *20, *22, *26). Moreover, no functional variants in *POR* or GABA_A_ receptor-encoding genes were identified. However, we detected the missense variant rs139927449 in *UGT1A4* (c.53T>A), which causes a Leu-to-His substitution at position 18 of the UGT1A4 precursor protein. This SNP is rare, with a minor allele frequency of only 0.00018 in the European population. The p.Leu18His polymorphic variant of UGT1A4 occurs within the signal peptide, 11 amino acids upstream of the cleavage site and the start of the mature protein. A bioinformatic analysis using SignalP 4.1 software indicated that the UGT1A4 p.Leu18His variant is associated with a marked decrease in the probability of signal peptide cleavage (41% vs. 83% of the wild-type UGT1A4 precursor protein). As shown for the more common p.Pro24Thr (*UGT1A4* c.70C>T) variant, altered signal peptide cleavage may decrease the glucuronidation activity of the mutant *UGT1A4* [160].

In conclusion, this case of prolonged midazolam-associated sedation highlights the factors crucial to consider during evaluation. Patient-specific variables, such as obesity, renal dysfunction, or hepatic dysfunction, can contribute to an extended elimination half-life of midazolam. Additionally, significant causes of prolonged midazolam-associated sedation include drug–drug interactions resulting from medications co-administered with midazolam, which are known to inhibit the CYP3A4 enzyme system. These medications belong to classes such as azole antimycotics, protease inhibitors, calcium channel antagonists, and macrolide antibiotics. For example, drugs like erythromycin or ketoconazole have been shown to markedly increase the AUC of orally administered midazolam by 3-fold or 15-fold, respectively. In the absence of the aforementioned factors, pharmacogenetic testing may be warranted. This case example is the first description of the application of whole-exome sequencing to identify genetic susceptibility factors associated with an adverse reaction to midazolam. Our results suggest that a rare mutation in *UGT1A4* may be responsible for the decreased clearance of midazolam and its active metabolite 1-OH-M and thus for prolonged midazolam-associated sedation and respiratory depression. Because the metabolic pathways of benzodiazepines differ, it is possible that the patient could tolerate another benzodiazepine well. The functional relevance of the identified mutation, however, remains to be confirmed by experimental studies.

## 14. Conclusions

Although there is a correlation between the plasma concentrations of midazolam and its active metabolite 1-OH-M and the degree of sedation at the population level, predicting the extent of sedation in an individual based solely on these concentrations is impossible [161]. This unpredictability for individual patients stems from the high interpatient variability in midazolam pharmacokinetics. This review emphasizes the numerous factors influencing midazolam pharmacokinetics. The case example illustrates the complexity of identifying the cause of unexpected side effects of midazolam, often without reaching a definitive conclusion. Furthermore, the case highlights the issue of “missing heritability”, which is characteristic of pharmacogenetics involving CYP3A4.

## Data Availability

The data used to support the findings of the case report are available on request.
